# Diversity, distribution, and relative abundance of medium and large-sized mammals in Chukala Mountain Forest, East Shoa Zone, Oromia, Ethiopia

**DOI:** 10.1186/s40850-024-00207-x

**Published:** 2024-07-24

**Authors:** Nimona Alemu, Tsegaye Gadisa, Tadesse Habtamu, Tolera Kuma

**Affiliations:** 1 College of natural and Computational Sciences, Department of Biology, Dambi Dollo University, Dambi Dollo town, Oromia, Ethiopia; 2 College of natural Sciences, Department of Biology, Jimma University, Jimma town, Oromia, Ethiopia; 3 College of natural and Computational Sciences, Department of Biology, Ambo University, Ambo town, Ambo, Ethiopia

**Keywords:** Mammals distribution, Mammals’ diversity, Chukala Mountain forest, Mammals

## Abstract

This study investigates the diversity, distribution, and relative abundance of medium and large-sized mammals in the biodiverse Chukala Mountain Forest of East Shoa, Oromia, Ethiopia, during March to August 2020. Three distinct habitat types—Montane forest with grassland (Panthera pardus, Papio anubis), woodland (Lepus fagani), and riverine forest (Procavia capensis)—were surveyed using line transects. Over four months, surveys were conducted bi-monthly, focusing on parameters such as species richness, and population distribution. Analysis revealed twelve mammal species spanning five orders and eight families, with olive baboons (Papio anubis) prevailing as the most abundant species, while leopards (Panthera pardus), bush hares (Lepus fagani), and rock hyraxes (Procavia capensis) were less frequently encountered. Woodland habitats exhibited the highest species richness (H = 1.700), followed by montane forest with grassland (H = 1.156) and riverine forest (H = 1.070). Notably, montane forest with grassland and riverine forest habitats shared similar species compositions across seasons (SI = 1). In conclusion, these findings provide valuable insights into the mammalian diversity and ecology of the Chukala Mountain Forest, highlighting the importance of ongoing conservation efforts in the region. Based on the findings, it is recommended to implement conservation measures focusing on preserving and enhancing the habitats of less common species such as the leopard, bush hare, and rock hyrax. Continual monitoring and research are recommended to track population dynamics and guide conservation initiatives for long-term ecosystem preservation. Overall, this study emphasizes the importance of proactive conservation measures in maintaining the ecological integrity of this vital ecosystem.

## Introduction

Ethiopia’s remarkable geographical features, including extensive altitudinal variation, contribute to its rich biodiversity. This diversity, shaped by factors such as altitude, rainfall, and soil variability, fosters a wide array of plant and animal life [1]. The resulting ecological complexity influences various aspects of the country, from agriculture to wildlife distribution [2]. With an estimated 6,500 to 7,000 species of higher plants, including 15% endemics, Ethiopia boasts a diverse flora [3]. This ecological richness underscores the need for comprehensive conservation efforts to safeguard Ethiopia’s biological heritage.

Mammals are crucial components of global biodiversity, categorized by body weight into small, medium, and large species. Medium-sized mammals weigh between 2 kg and 7 kg and include various carnivores, primates, rodents, hyraxes, and pangolins, while those exceeding 7 kg are considered large-sized mammals, encompassing diurnal primates, most carnivores larger than a fox, and perissodactyls and artiodactyl [4, 5]. The functional traits of medium and large-sized mammals, such as feeding habits and activity patterns, are influenced by environmental factors like disturbance and resource availability [6]. These mammals play essential roles in ecosystem functioning, including pollinating plants, dispersing seeds, recycling nutrients, and regulating populations through predator-prey interactions [7, 8]. They also significantly impact vegetation structure and composition. Large mammals act as ecological engineers by altering the surrounding vegetation, while both medium and large-sized mammals have cascading effects on ecosystems beyond direct species interactions [9]. Due to their broader habitat requirements, large-sized mammals serve as valuable indicators of habitat health [10].

Ethiopia boasts rich faunal diversity, with 320 recorded mammalian species, including 39 endemics [11, 12]. Efforts to protect this biodiversity include designating conservation areas such as national parks, wildlife reserves, and sanctuaries. However, such actions can harm fauna by disrupting habitats, increasing human-wildlife conflicts, and reducing reproductive rates. Understanding mammalian diversity, distribution, and abundance is crucial for effective land management [13].

Local forest areas play a significant role in conserving diverse mammalian species in Ethiopia [14]. These forests provide essential habitats for various mammalian species, supporting their survival and reproductive success [15]. Furthermore, they serve as crucial corridors connecting fragmented habitats, facilitating the movement of mammalian populations and promoting genetic diversity [16]. By preserving these forested areas, Ethiopia can mitigate threats such as habitat loss and fragmentation, which are significant drivers of mammalian population declines [17]. This conservation effort not only helps maintain healthy ecosystems and biodiversity [11] but also ensures the continued existence of Ethiopia’s diverse mammalian fauna for future generations.

The Chukala Mountain Forest, situated in East Shoa, Oromia, Ethiopia, serves as a crucial habitat for a diverse array of medium and large-sized mammals. Ethiopia’s remarkable biodiversity, shaped by its unique geography and ecological variability, underscores the importance of understanding mammalian populations within the region [15–17]. However, threats such as habitat loss and conflicts pose significant challenges to this biodiversity [14]. By leveraging recent advancements in field surveys and ecological modeling techniques [17], this research aims to unravel the complexities of mammalian communities in the region. Through comprehensive data collection and analysis, our findings will contribute valuable insights to inform conservation strategies aimed at preserving Ethiopia’s rich mammalian heritage and promoting the sustainable management of its ecosystems.

Given its ecological significance and conservation concerns, the Chukala Mountain Forest emerges as a key area for research [18]. Recent studies underscore the importance of understanding mammalian diversity and distribution in forest ecosystems like the Chukala Mountain Forest, located in East Shoa Zone, Oromia, Ethiopia [19, 20]. This region is known for its diverse habitats, including montane forests, woodlands, and riverine areas, which support a variety of medium and large-sized mammalian species [21]. However, comprehensive studies on their abundance and habitat preferences are scarce. This research aims to fill this gap by assessing mammalian populations across different habitats and seasons, contributing vital insights for biodiversity conservation and sustainable management in this ecologically significant area.

Recent works further emphasize the need for updated and detailed studies in this region. For example [22], examined mammalian diversity and habitat preferences in Bale Mountains National Park, and [23] studied the effects of habitat fragmentation on large mammals in the Ethiopian Highlands. Additionally [24], highlighted the impact of land use change on mammalian diversity in Eastern Ethiopia, while [25] assessed the conservation status of endemic mammals in Ethiopian protected areas. The study [26] explored the role of connectivity of forest patches in conserving medium and large-sized mammals in Western Ethiopia.

Despite its recognized importance, there remains a gap in our knowledge regarding the specific composition, distribution, and abundance of mammal species within the forest. To address this gap, this study aims to investigate the diversity, distribution, and relative abundance of medium and large-sized mammals in the Chukala Mountain Forest.

## Materials and methods

### Description of study area

**Fig. 1:**
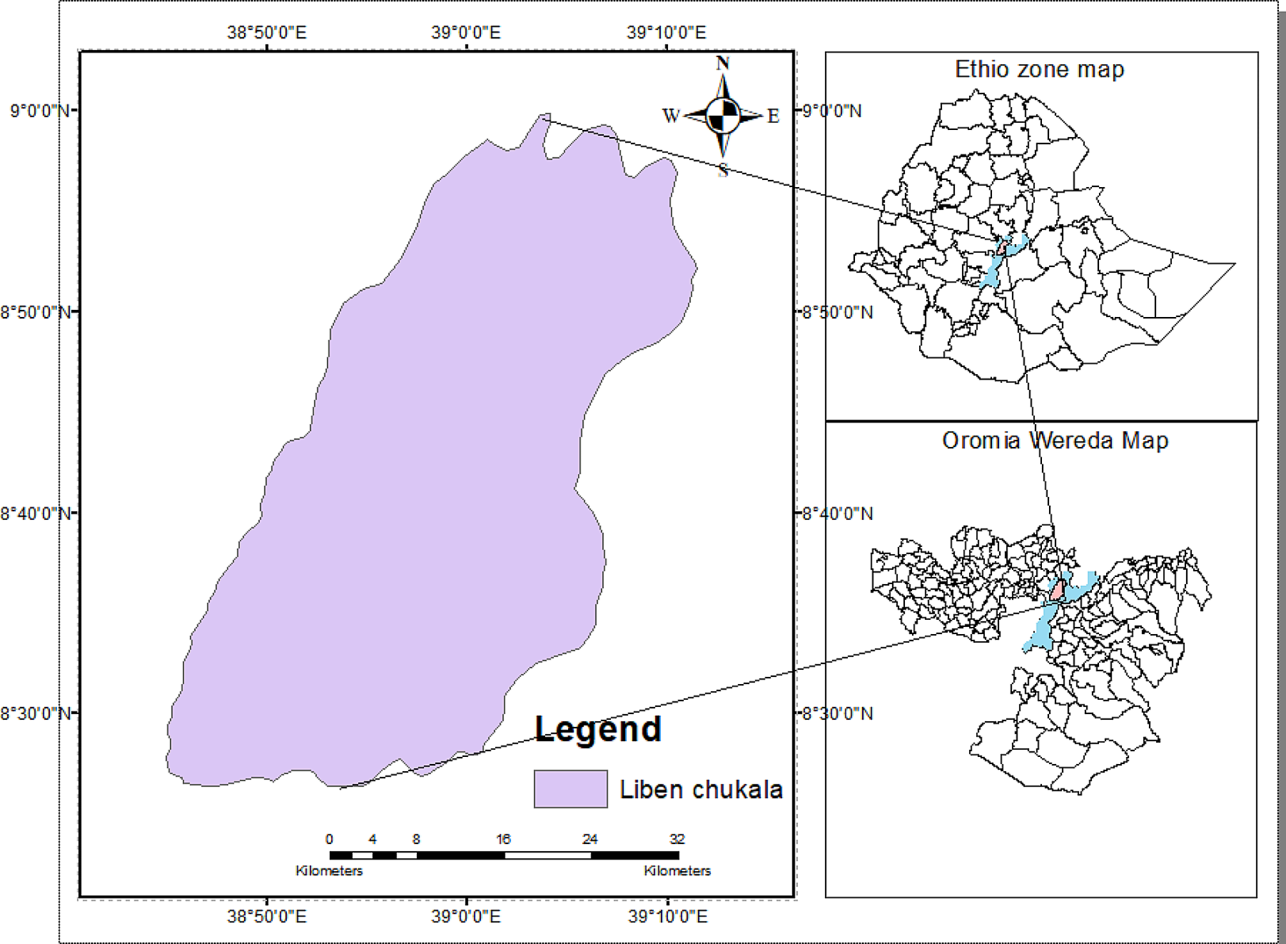
Map of the study area

The Chukala Mountain Forest, located in the Liben Chukala Woreda of the Eastern Shoa Zone in the Oromia National Regional State, has been recognized since ancient times under various names such as Zuquala, Chukala, and Zeqwala. Positioned at approximately 8.5500° *N* latitude and 38.8670° E longitude, its peak stands as a significant landmark [14]. Covering a total area of 9629 hectares, it comprises three of the 19 Kebeles in the Woreda, with the town of Adulala serving as its capital. Atop the mountain rests the Abo Gedam monastery, featuring a sacred crater lake. Topographically, the mountain reaches an estimated altitude of around 3000 m above sea level, with an Afro-mountain forest covering its peak, dominated by Juniperus procera trees. Despite its significance, physical infrastructure development on the mountain and its surroundings remains minimal compared to other regions in Ethiopia (Fig. 1). The mountain’s summit was once lush with a sprawling forest extending to the edge of the Weina Dega agroclimatic zone. Now, this forest is limited to the area around Crater Lake, covering about 100 hectares. Rich biodiversity thrives here, thanks to the altitude ranging from 1720 to 2989 m above sea level. The lake is surrounded by vast grasslands, but springs are drying up. Dominant tree species include Juniperus procera, Podocarpus gracilior, and Olea africana [15]. These forests, with their diverse flora, encircle the stunning Crater Lake atop a grassy mountain. While the area is rich in water sources, grass for mammals is scarce due to the water’s distribution. Despite challenges, the forests maintain diverse flora, varying with altitude and soil conditions.

### Preliminary survey

Before initiating the main study, a five-day exploratory survey took place in Chukala Mountain Forest from March 1 to 5, 2020. The primary objective was to categorize the research area into distinct habitat types based on vegetation and altitude. This pre-survey provided valuable insights into the forest’s topography, wildlife, plant diversity, and climate. Utilizing prevalent vegetation covers and altitude variations, sampling locations were selected and classified into various habitat types. In most instances, an initial survey was conducted as an integral part of the research protocol.

### The sampling design and data collection

#### Sampling design

In the initial fieldwork, researchers conducted a meticulous preliminary survey aimed at gaining a profound understanding of the study area’s characteristics [15]. This comprehensive investigation delved into various factors, including vegetation type and topography, to meticulously delineate distinct habitat zones. These zones, identified as Montane Forest with Grassland, Riverine forest, and woodland, were meticulously categorized to represent unique ecological niches within the research area [23].

To ensure the accuracy of mammal species abundance estimation, particularly focusing on large and conspicuous species, researchers employed the robust methodology of line transects [14]. Line transects have long been established as one of the most reliable methods for such assessments, particularly in open habitats where mammal sightings are easily observable. Furthermore, the utilization of line transects facilitates the estimation of abundance indices such as dung piles and burrows, providing valuable insights into mammal populations [15].

To mitigate any potential biases and ensure precise data collection, transect lines were strategically spaced approximately 0.5 to 1 km apart [23]. This systematic approach allowed for comprehensive coverage of the study area while minimizing the risk of overlapping observations. Moreover, the dimensions and placement of transects were meticulously tailored based on various factors, including topography, vegetation cover, and the size of habitat types [15]. In total, researchers established two lines transects for each of the three habitat types, resulting in a total of six transect lines meticulously distributed across the research area [23]. This rigorous and systematic approach not only facilitated comprehensive coverage of the diverse habitats but also enabled precise estimation of mammal abundance. By integrating the line transect methodology with meticulous habitat delineation, researchers aimed to ensure the reliability and robustness of abundance estimates for medium and large mammal populations within the study area.

#### Data collection

The investigation of the medium- and large-sized mammal population in the research area involved walking along randomly selected line transects. Observers recorded sightings of animals on both sides of the transect lines, either directly with the naked eye or using binoculars. It’s important to note that some animals adjacent to the path may go unnoticed, and the assumption is that all animals on the path are observed [14]. For nocturnal and naturally rare species, indirect indicators such as droppings, tracks, hair, vocal sounds, quills, and other signs along the transect line were utilized to infer species presence [15].

Data collection occurred across two seasons: the dry and rainy seasons. Wet season data were gathered in July and August of 2020, while dry season data were collected in March and April of 2020. Each transect line was surveyed twice in every habitat type during each season. Observers were positioned on both sides of the trail to ensure comprehensive coverage. Surveys were conducted between 06:00 and 10:00 am and between 16:00 and 18:00 in the late afternoon, aligning with peak mammal activity periods in the study area [23].

Species identification was based on various references, including the Field Guide to African Mammals [27], digital sources, and consultations with local indigenous people for their knowledge of species names. Field identification relied on visible morphological characteristics such as body size, coloration, and the structure of organs like tails and ears. To minimize disturbance to animals, observers aimed to reduce noise levels and move against the wind direction whenever possible. Additional information on climate and population dynamics was obtained from relevant organizations.

Data gathering took place over consecutive fifteen-day periods in each season, ensuring thorough coverage and accounting for potential variations in mammal activity and behavior. Typically, a standard approach involves allocating at least 15 to 20 field days per season for mammal surveys, considering factors like habitat size, accessibility, and logistical constraints [15]. This allocation is crucial as solely conducting a pair of trips to each habitat per season may not be adequate for comprehensively studying medium and large-sized mammals. Additional field trips and sampling occasions could provide a more comprehensive understanding of their behaviors and population dynamics.

#### Data analysis

Researchers investigating mammal diversity in the Chukala Mountain Forest employed various methods to quantify and categorize species presence [28]). One key metric was species diversity, which considers both the number of species present (richness) and their relative abundance within the ecosystem. To assess this, they utilized the Shannon-Wiener Index (H’) [29]). This index assigns a higher value to communities with a greater number of species and a more even distribution of individuals among those species. The formula for Shannon-Wiener Index is:

$${H}^{{\prime }}=-{\sum }_{n=1}^{S}(\text{p}\text{i}\text{}\cdot \text{l}\text{n}(\text{p}\text{i}\text{}\left)\right)$$

where H’ is the diversity index, S is the number of species, pi is the proportion of individuals belonging to a particular species (i^th^ ), and ln is the natural logarithm as outlined in Magurran, [29]).

Another important aspect of diversity is evenness, which reflects how equitably individuals are distributed among the various species. Pielou’s Evenness Index (J) was employed to quantify this [30]). This index approaches a value of 1 when all species have similar abundance, and deviates downward when certain species are significantly more abundant than others. The formula for Pielou’s Evenness Index is.

$$\text{J}=\frac{H{\prime }}{ln\left(S\right)},$$

where J is the evenness index, H’ is the Shannon-Wiener Diversity Index (previously calculated), and S is the number of species [30]).

To determine how similar the mammal communities were between seasons and across different habitats, researchers used Simpson’s Similarity Index (SI) [31]). This index provides a value between 0 (no similarity) and 1 (complete similarity) based on the proportion of species shared by the two communities being compared. The formula for Simpson’s Similarity Index is $\text{S}\text{I}=\frac{2C}{(A + B)},$ where SI is the similarity index, C is the number of species common to both habitats (A and B), and A and B represent the total number of species observed in each respective habitat [31]).

Lastly, researchers calculated the abundance of each mammal species. This was achieved by following formula.

$$\begin{array}{l}{\rm{abundance}} = \\\frac{{total\,number\,of\,individuals\,counted\,for\,a\,particular\,species}}{{the\,number\,of\,sampling\,blocks\,used\,in\,the\,study}}\end{array}$$

as used in the study [32]).

In addition to the quantitative measures, the researchers also established a classification system for mammal occurrence based on the frequency of sightings or signs during surveys [24]). This system categorized species as abundant (observed in over 75% of surveys), common (observed in 51–75% of surveys), frequent/fairly common (observed in 50% of surveys), uncommon (observed in 25–49% of surveys), or rare (observed in less than 25% of surveys) [28]).

By employing these various methods, researchers were able to gain a comprehensive understanding of the mammal community within the Chukala Mountain Forest. The combination of diversity indices, similarity measures, abundance calculations, and occurrence classifications provided valuable insights into the richness, evenness, and distribution of mammal populations across different habitats and seasons.

## Results

### Species composition

In Chukala Mountain Forest, a total of 794 mammal individuals belonging to 12 species, 8 families, and 5 orders were recorded during the current study, with 337 individuals during the dry season and 457 individuals during the wet season. Only three of these species—the rock hyrax (*Procavia capensis*), the bush hare (*Lepus fagani*), and the vervet monkey (*Chlorocebus aethiopis*)—were deemed medium-sized mammals; the other species were all large mammals. Out of all the families documented, Cercopitheci has made the largest contribution with three species. Bovidae and Suidea have also provided two species each, while the remaining four families—Leporidae, Felidae, Procaviidae, Hyaenidae, and Canidae—have one species each ( Table 1).

**Table 1:** Medium and large-sized mammalian species recorded from Chukala Mountain Forest, Oromia, Ethiopia

Order	Family	Common Name	Scientific Name	Afaan Oromo
Primate	Cercopitheci	Colobus monkey	*Colobus abyssinicus*	Weenni
		Vervet monkey	*Chlorocebus aethiopis*	Qamalee
		Olive baboon	*Papio anubis*	Jaldeessa
Lagomorpha	Leporidae	Bush hare	*Lepus fagani*	Illeetti
Hyracoidea	Procaviidae	Rock hyrax	*Procavia capensis*	Osolee
Artidactyla	Bovidae	Common bushbuck	*Traglaphus scriptus*	Borofa
		Grey Duiker	*Sylvicapra grimmia*	Kuruphe
	Suidae	Bushpig	*Potamochoerus larvatus*	Booyyee
		Warthog	*Phacochoerus africanus*	Karkarro
Carnivora	Hyaenidae	Striped hyena	*C.carcuta*	Waraabessa
	Felidae	Leopard	*Panthera pardus*	Qeerransa
	Canidea	Common jackal	*Canis aereus*	Jeedala/sardiida

#### Diversity indices and evenness of medium and large-sized mammals

In the dry season, woodland had the highest animal diversity (H = 1.700), while riverine forest was the least diversified habitat (H = 1.070), and montane forest with grassland was the second most diversified (H = 1.156). During this season, the estimated species evenness for woodland, montane forest with grassland, and riverine forest was J = 0.709, J = 0.646, and J = 0.597, respectively.

During the wet season, woodlands had the highest recorded diversity (H = 1.575). The least diverse habitat during that season was riverine forest (H = 1.105), while the second-most diversified environment was montane forest with grassland (H = 1.115). Species evenness values for grassland, woodland, and erica forest for this season were J = 0.622, J = 0.634, and J = 0.687, respectively (Table 2).

**Table 2:** Diversity indices of medium and large-sized mammals in different Habitat types during dry and Wet Seasons

Habitat Type	No. of Species	No. of Individuals	SWI (H’)	H’ max	Evenness (J)
MF with Grassland	6	218	1.151	1.792	0.642
Woodland	12	359	1.375	2.398	0.553
Riverine Forest	6	207	1.093	1.79	0.610

#### Distribution of mammals observed in different habitat types

The 12 documented mammal species were discovered through indirect and direct observation methods. There were differences in the distribution of animal species among the three types of habitat and seasons. Colobus monkeys and leopards were observed throughout the montane forest, which has grassland habitat in all seasons and woods in the wet season. Vervet monkeys were seen in habitats such as woodlands and riverine forests in both dry and wet seasons. Olive baboons, the dominant mammalian species in the research area, were observed in three habitats in both seasons. Common bushbucks, grey duikers, and spotted hyenas were observed in all habitats in both seasons. Certain species, such as the bush hare, rock hyrax, bush pig, and warthog, were only observed in forest habitats during both seasons. Common jackals were observed in both woodland and riverine forest habitats in both dry and wet season (Table 3) .

**Table 3:** Distribution of medium and large-sized mammals along Study habitats in the study area

Species	Species Identification Method	Habitat Type
Colobus monkey	Visual	Montane Forest with Grassland (Dry), Woodland (Wet)
Vervet monkey	Visual	Woodland (Dry and Wet), Riverine Forest (Dry and Wet)
Olive baboon	Visual	Montane Forest with Grassland (Dry and Wet), Woodland (Dry and Wet), Riverine Forest (Dry and Wet)
Bush hare	Visual	Woodland (Dry and Wet)
Rock hyrax	Visual	Woodland (Dry and Wet)
Common bush buck	Visual/dropping/footprint	Montane Forest with Grassland (Dry and Wet), Woodland (Dry and Wet), Riverine Forest (Dry and Wet)
Grey Duiker	Visual/dropping/footprinting	Montane Forest with Grassland (Dry and Wet), Woodland (Dry and Wet), Riverine Forest (Dry and Wet)
Bushpig	Visual	Woodland (Dry and Wet)
Warthog	Feaces/Visual	Woodland (Dry and Wet)
Spotted hyena	Feaces/Visual	Montane Forest with Grassland (Dry and Wet), Woodland (Dry and Wet), Riverine Forest (Dry and Wet)
Leopard	Indirect/drop	Montane Forest with Grassland (Dry and Wet), Woodland (Dry and Wet)
Common jackal	Visual	Woodland (Dry and Wet), Riverine Forest (Dry and Wet)

### Occurrences of mammals

The medium- and large-sized mammals were classified as common, uncommon, or rare based on their occurrence in the study area. The 12 mammalian species identified in the research area were divided into 33.33% common, 25% uncommon, and 41.67% rare (Table 4).

**Table 4:** Occurrence of medium and large-sized mammals in the study area

Common	Uncommon	Rare
Grey Duiker	Colobus monkey, Vervet monkey	Warthog
Spotted hyena	Leopard	Common jackal
Olive baboon		Bush hare
Common bush buck		Rock hyrax
		Bush pig

### Relative abundance of mammals

Among the 12 species of mammals recorded, the Olive baboon (*P. anubis*) was the most abundant species, contributing 53.64% and 56.46% of individuals during the dry and wet seasons, respectively. The Vervet monkey (*C. aethiopis*) was the second most abundant mammal, with 17.49% and 12.47% during the dry and wet seasons, respectively. Colobus monkey (*C. abyssinicus*) and Spotted hyena (*C. carcuta*) were the next most abundant species during the wet and dry seasons, respectively, with other species like Leopard (*P. pardus*), Bush hare (*L. fagani*), and Rock hyrax (*P. capensis*) being the least diversified.

Seasonal variations were observed in the mammalian species composition and the number of individuals among habitats and between seasons. The highest number of individuals of medium and large-sized mammals were recorded in the Woodland habitat (254), followed by Montane Forest in the Grassland habitat (128) and the Riverine forest with 74 during the wet season. More individuals were also observed in the Riverine forest (133) than in woodland (105) and Montane Forest with Grassland (90) during the dry season. Within habitat, the seasonal abundance of mammals significantly varied for all habitats.

Seasonal variations were observed in the mammalian species composition and the number of individuals among habitats and between seasons (See Tables 5 and 6). The highest number of individuals of medium and large-sized mammals were recorded in the Woodland habitat (254), followed by Montane Forest in the Grassland habitat (128) and the Riverine forest with 74 during the wet season (Table 7). More number of individuals is also observed in the Riverine forest (133) than in woodland (105) and Montane Forest with Grassland (90) during the dry season (Table 5). Within habitat, the seasonal abundance of mammals was significantly varied for all habitats (Montane Forest with grassland: χ2 = 10, 1 df, *P* < 0.05; Woodland: χ2 = 1.33, 1 df, *P* < 0.05 and Riverine forest: χ2 = 12.5, 1 df, *P* < 0.05).

**Table 5:** The relative abundance of medium and large-sized mammalian species recorded in different habitat types during both dry and wet seasons

Species	Habitat Type	Dry Montane Forest with Grassland	Wet Montane Forest with Grassland	Dry Woodland	Wet Woodland	Dry Riverine Forest	Wet Riverine Forest
Colobus monkey	Abundance (%)	30	36	-	16	-	-
	Total Individuals	-	-	-	-	-	-
Vervet monkey	Abundance (%)	-	-	32	18	25	42
	Total Individuals	-	-	32	18	25	42
Olive baboon	Abundance (%)	78	42	142	60	38	60
	Total Individuals	78	42	142	60	38	60
Bush hare	Abundance (%)	-	-	-	1	3	-
	Total Individuals	-	-	-	1	3	-
Rock hyrax	Abundance (%)	-	-	1	2	-	-
	Total Individuals	-	-	1	2	-	-
Common bushbuck	Abundance (%)	9	4	10	2	5	2
	Total Individuals	9	4	10	2	5	2
Grey Duiker	Abundance (%)	3	1	12	4	1	3
	Total Individuals	3	1	12	4	1	3
Bushpig	Abundance (%)	-	-	12	5	-	-
	Total Individuals	-	-	12	5	-	-
Warthog	Abundance (%)	-	-	7	4	-	-
	Total Individuals	-	-	7	4	-	-
Spotted hyena	Abundance (%)	7	5	13	10	8	6
	Total Individuals	7	5	13	10	8	6
Leopard	Abundance (%)	1	2	2	1	-	-
	Total Individuals	1	2	2	1	-	-
Common jackal	Abundance (%)	-	-	6	6	-	-
	Total Individuals	-	-	6	6	-	-
Total	Total Individuals (All Species)	128	90	254	105	74	133

**Table 6:** Relative abundance of medium and large-sized mammal species recorded in different habitat types during both dry and wet seasons

Species	Habitat Type	Dry Grassland	Wet Grassland	Dry Woodland	Wet Woodland	Dry Riverine Forest	Wet Riverine Forest
Colobus monkey	Relative Abundance (%)	36	40	30	23.62	0	0
Vervet monkey	Relative Abundance (%)	-	-	18	17.14	32	12.6
Olive baboon	Relative Abundance (%)	42	46.66	78	60.42	60	57.14
Bush hare	Relative Abundance (%)	-	-	3	2.86	1	0.39
Rock hyrax	Relative Abundance (%)	-	-	2	1.91	1	0.39
Common bushbuck	Relative Abundance (%)	4	4.49	9	7.03	2	1.91
Grey Duiker	Relative Abundance (%)	1	1.11	3	2.34	4	3.81
Bushpig	Relative Abundance (%)	-	-	5	4.76	12	4.72
Warthog	Relative Abundance (%)	-	-	4	3.81	7	2.76
Spotted hyena	Relative Abundance (%)	5	5.55	7	5.47	10	9.52
Leopard	Relative Abundance (%)	2	2.22	1	0.78	1	0.95
Common jackal	Relative Abundance (%)	-	-	6	5.71	6	2.36
Total	Total Individuals	90	100	128	100	105	100

**Table 7:** Comparison of medium and large-sized mammal species similarity between habitat types during combined seasons

Habitat Types	Montane Forest with Grassland	Riverine Forest
*Woodland (Wet Season)*	*0.667*	*0.706*
*Woodland (Dry Season)*	*0.667*	*0.706*
*Riverine Forest (Wet Season)*	*0.91*	*1*
*Riverine Forest (Dry Season)*		

## Discussion

The Chukala Mountain Forest in Oromia, Ethiopia, harbors a unique assemblage of twelve medium and large-sized mammal species, distinct from other regions in the country [33]. This biodiversity hotspot, despite its central location within a densely populated area [34], faces challenges due to human activities [32]. Dry season brings a peak in mammalian diversity for riverine forests and woodlands due to their proximity to water sources [35, 36]. However, limited water flow during this period remains a concern for sustaining mammal populations [36]. Primate species like the Olive baboon, Vervet monkey, and Colobus monkey are particularly dominant in the area, highlighting their ecological significance and the need for their conservation [37].

Woodland habitats hold the highest mammalian diversity during both wet and dry seasons, with riverine forests showing the least [38, 39]. This emphasizes the influence of habitat characteristics on species abundance. The Chukala Mountain Forest’s significance as a biodiversity sanctuary is undeniable, housing a unique array of twelve medium and large-sized mammal species within its ecosystem [34]. This underscores the importance of prioritizing conservation efforts in this area. Human activities such as agriculture, settlement, and infrastructure development pose significant threats to the forest’s ecological balance and the survival of its wildlife inhabitants [32]. Sustainable land management practices and habitat restoration initiatives are crucial for addressing these challenges. Preserving critical habitats like riverine forests and woodlands is essential to ensure the year-round availability of water and food resources for wildlife [36].

Protecting keystone species such as the Olive baboon, Vervet monkey, and Colobus monkey is paramount for maintaining the forest’s ecosystem health [37]. These species play a vital role in ecosystem functioning, and their conservation is critical for preserving overall biodiversity. Collaborative conservation strategies, including the establishment of wildlife corridors and engagement with local communities, are instrumental in enhancing habitat connectivity and promoting sustainable biodiversity conservation in the Chukala Mountain Forest [40].

By prioritizing the preservation of this unique ecosystem and its diverse wildlife populations, we can ensure its long-term sustainability and contribute to the broader conservation efforts in the region. This study highlights the urgent need for proactive measures to safeguard the Chukala Mountain Forest and underscores its significance as a valuable asset for biodiversity conservation. Conservation efforts must prioritize maintaining critical habitats like riverine forests and woodlands to ensure the availability of resources for wildlife [32]. Sustainable land management practices are essential to mitigate human-induced threats such as habitat loss and fragmentation. By safeguarding these ecosystems and engaging local communities in conservation initiatives, the unique biodiversity of the Chukala Mountain Forest can be preserved for future generations.

## Conclusion and implications for conservation

### Conclusion

Throughout the research conducted in the Chukala Mountain Forest, a total of 12 species of medium- and large-sized mammals were meticulously identified and documented. Among these species, nine were classified as large mammals, while three were considered medium-sized. Seasonal variations in mammal distribution and abundance across different habitats within the forest were observed. During the dry season, comparable species richness was noted between Montane forests with grassland and Riverine forests, with woodlands exhibiting the highest species diversity. Conversely, during the rainy season, the woodland habitat harbored the highest number of individuals, while the riverine forest had the fewest. These findings underscore the dynamic nature of mammalian populations in response to seasonal changes in the Chukala Mountain Forest.

In conclusion, the study highlights the significance of understanding seasonal variations in mammalian abundance and distribution for effective conservation management in the Chukala Mountain Forest. The observed fluctuations in species composition and individual counts between dry and rainy seasons emphasize the importance of adaptive conservation strategies. By incorporating these findings into conservation planning, we can better preserve the biodiversity of this unique ecosystem and ensure the long-term sustainability of mammal populations within the Chukala Mountain Forest.

### Recommendations

Based on the study in Chukala Mountain Forest, precise recommendations include:

i. Influence policymakers to prioritize biodiversity conservation, sustainable land management, and community livelihoods in African montane forests.
ii. Enhance sustainable ecotourism to benefit local communities and raise conservation awareness effectively.
iii. Foster collaborations among institutions to advance understanding of mammalian ecology and conservation in montane forests.
iv. Engage local communities in wildlife monitoring and sustainable land practices to enhance conservation efforts.
v. Create pathways between habitats to facilitate mammalian movement and genetic diversity.
vi. Develop strategies to manage water in riverine habitats during dry seasons to support mammalian populations.

### Limitation and future directions

The study impressively investigates mammal diversity, distribution, and relative abundance in the Chukala Mountain Forest. However, the sampling methods, confined to a few line transects per habitat, offer room for refinement. Future research could expand sampling techniques, incorporating approaches such as camera trapping, and involve local communities to bolster conservation efforts. These enhancements promise to advance our understanding of mammal ecology in the region. Moreover, fostering engagement with local communities can amplify data collection and conservation efforts, paving the way for more impactful strategies. Through these proactive measures, future studies can build upon the existing foundation and significantly contribute to advancing our knowledge of mammal ecology in the region.

## Data Availability

All the data were presented in the manuscript.
